# Autophagy supports the storage of lytic granules in human NK cells

**DOI:** 10.1038/s41419-026-08937-1

**Published:** 2026-06-01

**Authors:** Piera Filomena Fiore, Sergio Forcelloni, Simone Vitozzi, Nicola Tumino, Tobias Theinert, Lokossou William Sanvi, Francesca Nazio, Valentina D’Oria, Stefania Martini, Maximilian Reichert, Lorenzo Moretta, Ignazio Caruana, Paola Vacca

**Affiliations:** 1 Innate Lymphoid Cells Unit, Immunology Research Area, Bambino Gesù Children’s Hospital, IRCCS, Rome, Italy; 2 Laboratory for Cell and Gene Therapy, Department of Pediatrics, Hematology, Oncology, Stem Cell Transplantation and Cell Therapy, University Hospital Würzburg, Würzburg, Germany; 3 Department of Pediatrics, Hematology, Oncology, Stem Cell Transplantation and Cell Therapy, University Hospital Würzburg, Würzburg, Germany; 4 Department of Biology, University of Rome Tor Vergata, Rome, Italy; 5 Core Research Facilities, Bambino Gesù Children’s Hospital, IRCCS, Roma, Italy; 6 UOC Patologia e immunologia Sperimentale, IRCCS Azienda Ospedaliera Metropolitana, Genova, Italy; 7 Translational Pancreatic Cancer Research Center, Medical Clinic and Polyclinic II, Klinikum rechts der Isar, Technische Universität München, Munich, Germany; 8 Tumor Immunology Unit, Bambino Gesù Children’s Hospital, IRCCS, Rome, Italy

**Keywords:** Innate lymphoid cells, Macroautophagy

## Abstract

Natural Killer (NK) cells are innate lymphoid cells that play an important role in immune defense against pathogens and tumors. Understanding the mechanisms that enhance NK cell effector functions could significantly improve current NK cell-based therapies. Autophagy is a lysosome-dependent degradation process essential for NK cell development and function. This study analyzed the autophagic potential of mature NK cell subsets in peripheral blood. We demonstrated that exposure to inflammatory cytokines reduces autophagy via mTOR signaling pathway. Specifically, the activation of NK cell receptors leads to a temporary decrease in autophagy, which is rapidly restored, demonstrating the dynamics of autophagy in response to activating signals. Importantly, continuous overexpression of key autophagy regulators significantly increased autophagic flux, which directly correlated with enhanced cytotoxicity and metabolic activity in NK cells. The increased cytotoxicity was supported by a greater accumulation of cytolytic granules and their associated proteins. Our findings indicate that activating stimuli reduce autophagy, whereas sustained autophagic activity under steady-state conditions is crucial for the formation and maintenance of cytolytic granules, supporting the persistent cytotoxic function of NK cells.

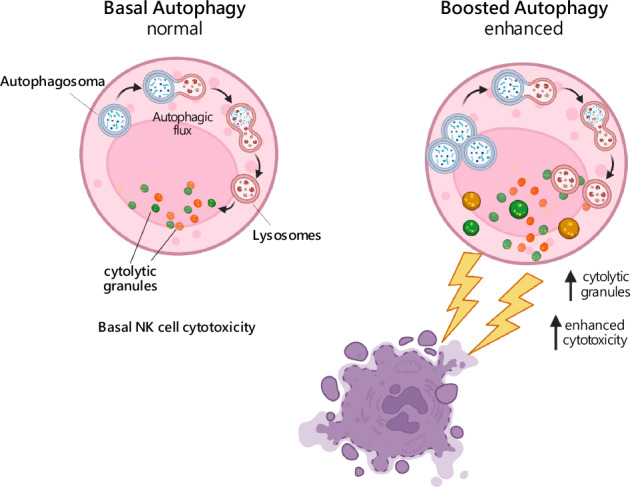

## Introduction

Natural Killer (NK) cells are innate lymphocytes characterized by their intrinsic ability to recognize and eliminate both virus-infected and tumor cells. Their cytotoxic activity is tightly regulated by a dynamic balance between activating and inhibitory cell-surface receptors, thereby allowing discrimination between target and healthy “self” cells [1]. Serving as a critical first line of defense, NK cells survey host tissues and respond rapidly to cellular stress signals. Furthermore, NK cell activity is fine-tuned by cytokines, including IL-2, IL-12, IL-15, and IL-18, which modulate their activation, proliferation, and cytotoxic potential [2].

In clinical oncology, NK cell-based immunotherapy has garnered significant attention due to its capacity to mediate potent antitumor responses while reducing the risk of graft-versus-host disease. However, the efficacy of this approach is profoundly challenged by the hostile tumor microenvironment (TME), which establishes a highly suppressive niche. This suppression is mediated by the expression of immune-checkpoint ligands and the release of inhibitory soluble factors, notably Transforming growth factor beta (TGFβ), Indoleamine-2,3-Dioxygenase (IDO), and Prostaglandin E2 (PGE2) [3, 4]. Moreover, TME stressors, such as hypoxia, nutrient depletion, acidosis, and abnormal vasculature, negatively affect NK cell functions, reducing their metabolic activity, cytotoxic capacity, and ability to infiltrate tumors. Overcoming these suppressive factors is essential for improving cancer treatment, necessitating strategies such as ex vivo pre-conditioning with cytokines/small-molecule drugs or engineering with chimeric antigen receptors (CAR) [5]. Therefore, achieving a deeper understanding of the key molecular pathways governing NK cell homeostasis and function is paramount to enhancing their therapeutic effectiveness.

A fundamental pathway influencing cellular fitness is autophagy, a self-degradation process that enables cells to recycle nutrients and maintain metabolic balance by degrading cytoplasmic components through lysosomal pathways. Autophagy is crucial for maintaining cellular homeostasis under various stress conditions, including nutrient deprivation, hypoxia, cell differentiation, and activation [6]. While autophagy has been shown to regulate immune responses, its role in mature human NK cell biology remains unclear. Autophagy is indispensable during NK cell development, particularly during the transition from progenitor to immature NK cells. Intriguingly, as NK cells mature, autophagic activity tends to decrease [7–9]. This decline may influence their functional capabilities and overall responsiveness in immune surveillance. Recent findings suggest that TME dysregulates autophagy in NK cells, thereby exacerbating mitochondrial dysfunction and reducing NK-cell infiltration and cytotoxicity in solid tumors [10]. However, the factors involved in regulating autophagy and the relationship between autophagy status and mature NK cell subsets still need further investigation.

A comprehensive and integrated understanding of autophagy dynamics in NK cells—both under homeostatic conditions and within the suppressive TME—may reveal novel strategies to enhance NK-cell metabolic fitness, persistence, and antitumor activity. Ultimately, unraveling these mechanisms could guide the rational design of therapeutics aimed at restoring or augmenting autophagy, thereby improving the efficacy of NK-cell–based immunotherapies in cancer.

In this study, we analyzed the transcriptional profiles of autophagy regulator genes in mature NK cell subsets to assess the relationship between autophagy status and NK cell phenotype. We also investigated autophagy flux in response to cytokine and activating receptor stimulation, as well as the impact of autophagy modulation on NK cell cytotoxic activity.

## Results

### Transcriptional profiling reveals no substantial difference in autophagy regulators across NK cell subsets from the peripheral blood of healthy individuals

Human peripheral blood (PB) NK cells can be broadly divided into two main subsets based on CD16 and CD56 expression: the CD56^bright^CD16^-^ subset, known for robust cytokine production and immunoregulatory function; and the more cytotoxic and abundant CD56^dim^CD16^+^ subset, which includes both conventional cytotoxic NK cells and adaptive NK cells [11–13]. Given that the ability to undergo autophagy is associated with the transcriptional regulation of autophagy-related genes [14, 15], we explored whether autophagy potential correlates with NK cell subset identity.

**Fig. 1:**
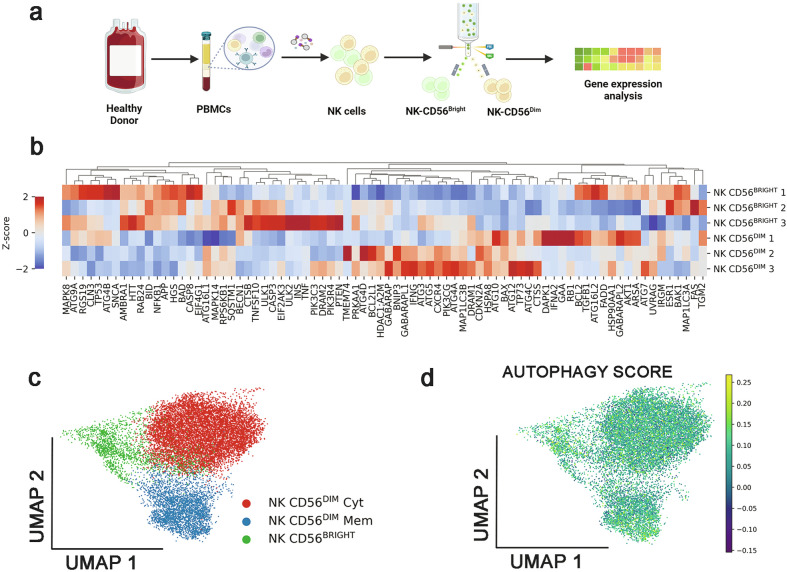
Transcriptional profile of autophagy regulators in mature human NK cell subsets from healthy human peripheral blood. **a** schematic overview of the experimental design workflow. **b** NK cells were isolated from the PB of HDs and sorted into CD56^dim^ and CD56^bright^ subpopulations. The expression of autophagy regulators was quantified by Real-Time PCR, with results shown in the heatmap below. The color scale represents Z-score normalized expression values for each gene. **c** Analysis of CITE-seq dataset of NK cells isolated from PBMCs of HDs. UMAP plot of NK cells subdivided into CD56^bright^, CD56^dim^ cytotoxic, and CD56^dim^ memory populations based on unsupervised clustering. **d** UMAP plot showing the autophagy score into CD56^bright^, CD56^dim^ cytotoxic, and CD56^dim^ memory populations.

Using cell-sorted NK cells from the PB of healthy donors (PB-HD), we compared the expression of autophagy regulators between the CD56^bright^CD16^-^ and CD56^dim^CD16^+^ subpopulations using real-time PCR (Fig. 1a). The results suggest a heterogeneous expression of autophagy genes, with no clear dominant autophagy profile among the subsets (Fig. 1b). To validate these findings, we examined publicly available high-dimensional CITE-Seq datasets of peripheral blood mononuclear cells (PBMCs) from eight healthy donors [11]. NK cells were identified based on the cell annotation by Hao et al. [11] and categorized into CD56^bright^, CD56^dim^ cytotoxic, and CD56^dim^ memory populations, following previous reports [13] (Figs. 1c and S1a, b). However, no differences were observed in the autophagy score among the three NK cell subsets (Fig. 1d). These cumulative results strongly suggest that the transcriptional profile of autophagy regulators is not intrinsically linked to, or strictly determinant of, the underlying autophagy potential within established mature NK cell subsets. These findings suggest that autophagy potential is not strictly determined by NK cell subset identity.

### IL-15-mediated activation reduces autophagic flux in NK cells via mTOR signaling

**Fig. 2:**
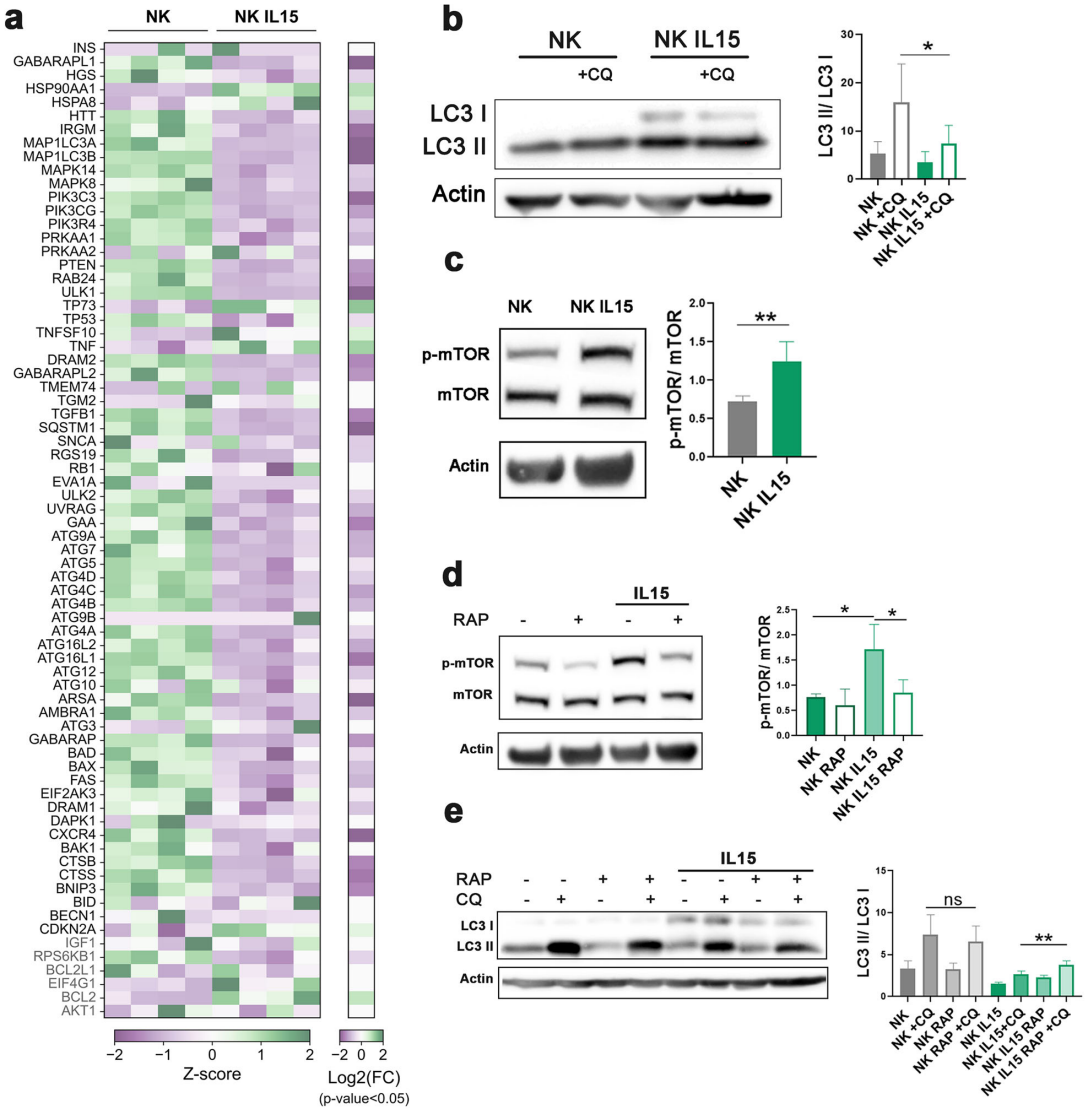
IL15-mediated activation decreases autophagy in mature NK cells. **a** Heatmap showing Real-Time PCR array analysis of autophagy-related genes in NK cells upon IL15 stimulation (*n* = 4 biological replicates per condition). Gene expression levels were normalized using Z-score transformation. Positive regulators of autophagy are indicated with black labels, while negative regulators are reported with gray labels. On the right, the log2 fold changes of autophagy-related genes between IL15-stimulated and unstimulated NK cells are shown (green: Log2(FC) > 0, purple: Log2(FC) < 0). Only statistically significant comparisons are included (paired t-test, *p* ≤ 0.05). **b** Representative Immunoblot and quantification of the LC3 II/LC3 I ratio changes in unstimulated and IL15-stimulated NK cells. NK cells were treated with 10 µM Chloroquine (CQ) before immunoblotting. **c** Representative Immunoblots and quantification of total and phosphorylated mTOR and ACTIN in unstimulated and IL15-stimulated NK cells. **d** Immunoblotting and quantification of total and phosphorylated mTOR and ACTIN in NK cells treated with 100 ng/ml of Rapamycin (RAP) in the presence or absence of IL15 for 24 h. **e** Levels of LC3 II/I and actin determined by western blotting in NK cells treated with 100 ng/ml of Rapamycin (RAP) in the presence or absence of IL15 for 24 h. **b**–**e** Data are presented as means ± SD of 4 to 6 biological replicates per condition. Statistical significance was assessed using a paired t-test (*p < 0.05, **p < 0.01).

Stress conditions, cytokines, growth factors, and receptor engagement trigger autophagy. To evaluate the impact of pro-inflammatory stimuli on autophagic flux, the expression of genes involved in the key steps of autophagy was analyzed in NK cells after cytokine stimulation. Thus, NK cells were isolated from PB-HD and activated with IL2 and IL15. After two weeks of activation and expansion, the cytokines were withdrawn, and NK cells were cultured with or without IL15 for an additional 24 h. The analysis of crucial autophagy regulators indicated that IL15 affected the expression levels of several autophagy regulators in NK cells (Fig. 2a). Moreover, IL15 stimulation reduces the conversion rates from LC3-I to LC3-II and enhances the activating phosphorylation of mTOR, an inhibitory factor of autophagy induction [16] (Fig. 2b, c). These effects were recapitulated in freshly isolated NK cells exposed to IL15, demonstrating that IL15–dependent autophagy regulation is an intrinsic feature of NK cell biology rather than a consequence of prolonged in vitro expansion (Fig. S2). To assess the role of mTOR signaling in the IL15-mediated regulation of autophagy flux, IL15-stimulated NK cells were treated with the mTOR inhibitor Rapamycin. After 24 h of treatment, a reduction of the mTOR phosphorylation (Fig. 2d) was detected. In addition, the decrease of the LC3-II/LC3-I ratio mediated by IL15 was partially prevented (Fig. 2e). These data suggest that IL15 modulates autophagy in NK cells by inducing the mTOR pathway. Previous studies have shown that IL-15 induces expression of BCL-2 family proteins via the PI3K/AKT pathway in T cells [17, 18]. Moreover, Bcl-2 binds Beclin-1, inhibiting its interaction with the class III phosphatidylinositol 3 kinase (PI3KC3) complex, which is critical for initiating autophagosome formation [19]. Similarly, we observed that IL-15 stimulation increases expression of Bcl-2 in NK cells (Fig. 2a and Fig. S3). Therefore, increased Bcl-2 expression may contribute to the negative regulation of autophagy mediated by IL15.

### NK cells restore basal levels of autophagy after cytotoxic activity

**Fig. 3:**
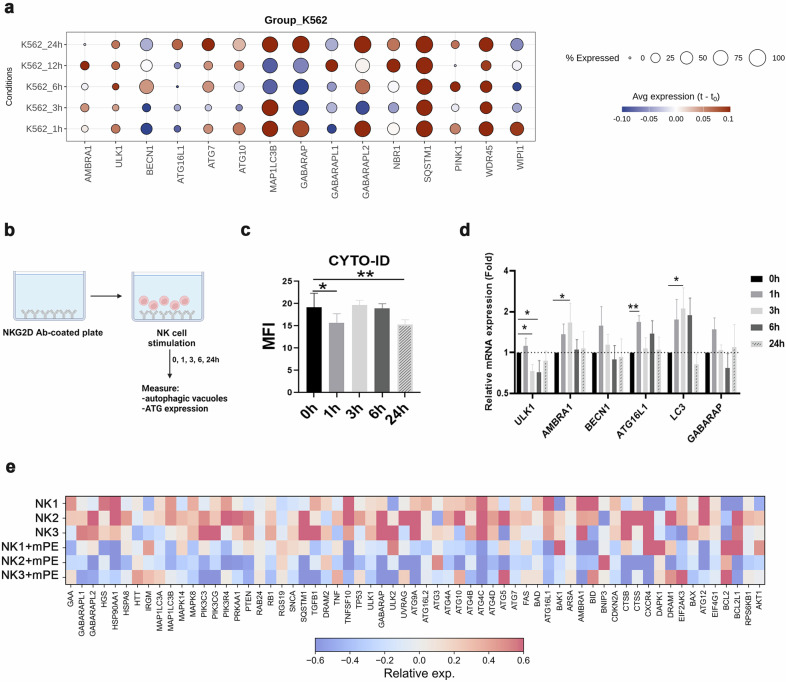
Autophagy regulation upon degranulation induction by activating receptor stimulation. **a** Custom dot plot showing the change in mean normalized expression (relative to the baseline, 0 h) of autophagy-related genes (ATG) in NK cells at different time points after co-culture with K562 leukemia cells. Color intensity represents the average change in normalized gene expression, while dot size indicates the percentage of cells expressing each ATG. **b** Schematic overview of the experimental design workflow. **c** Flow cytometry analysis of MFI CYTO-ID staining of NK cells stimulated or not with NKG2D. Data are presented as means ± SD of 4 biological replicates per condition. Statistical significance was assessed using a two-way Anova multiple comparisons (*p < 0.05, **p < 0.01). **d** Real-Time PCR analysis of ULK1, AMBRA1, BECN1, ATG16L1, LC3 and GABARAP transcripts normalized to 18S mRNA. Data are presented as means ± SD of 4 biological replicates per condition. Statistical significance was assessed using a two-way Anova multiple comparisons (*p < 0.05, **p < 0.01). **e** Heatmap showing protein expression in resting NK cells and NK cells stimulated with malignant pleural effusion (mPE). Values are z-score-normalized per gene across all donors and further centered within each donor pair (NK vs NK+mPE).

Tumor cells express a broad array of ligands for activating NK receptors, engagement of which recruits adaptor molecules and initiates downstream signaling cascades converging on the AKT–mTOR pathway [20]. To investigate the impact of simultaneous triggering of multiple activating NK receptors on autophagy regulation, we interrogated a publicly available scRNA sequencing (scRNA-seq) dataset profiling NK cells before and after co-culture with the K562 leukemia cell line, which expresses a wide repertoire of NK cell–activating. The results showed that exposure to target cells upregulated transcripts of key autophagy regulator genes (Fig. 3a), indicating that engagement of activating receptors is associated with transcriptional induction of autophagy-related genes.

These observations were not restricted to a single tumor model, as similar transcriptional changes were detected in NK cells cultured with additional tumor cell lines, including RI1, Jurkat, and GDM1 (Fig. S4), suggesting that induction of autophagy-related gene expression represents a general response of NK cells to tumor cell recognition.

Next, to validate the scRNA-seq analysis, we assessed the dynamics of autophagy modulation in NK cells during degranulation. Thus, NK cells were stimulated with the NKG2D monoclonal antibody and subsequently stained for autophagy vacuoles by CITO-ID at various time points (Fig. 3b). Quantitative analysis revealed that receptor engagement induced a rapid and transient reduction in autophagic vacuoles, consistent with an acute suppression of autophagic activity during the effector phase of NK cell activation. Notably, this initial decrease was followed by a progressive recovery of autophagy to basal levels (Fig. 3c), indicating a dynamic and reversible modulation of autophagy during NK cell cytotoxic responses.

In line with these functional observations, transcriptional analysis performed after degranulation showed increased expression of several core autophagy genes ULK1, AMBRA1, BECN1, ATG16L1, LC3 and GABARAP transcripts (Fig. 3d). This supports the induction of a compensatory transcriptional program aimed at restoring autophagic activity and cellular homeostasis following cytotoxic activity. Then, we assessed the expression of autophagy genes in response to tumor microenvironment soluble factors by exposing NK cells to the malignant pleural effusion (mPE) from lung cancer patients. After 24 h of incubation, NK cells showed decreased expression of several genes involved in autophagy, suggesting that prolonged exposure to the TME reduces NK cell autophagy potential (Fig. 3e).

Together, these results suggest that NK cell cytotoxic activity temporarily suppresses autophagy, followed by an increase in autophagy gene expression to restore homeostasis, while the soluble factors in the TME decrease in their expression. Moreover, NK cell cytotoxic engagement is characterized by a transient suppression of autophagy during the effector phase, followed by compensatory induction of autophagy-related gene expression to re-establish cellular homeostasis. In contrast, sustained exposure to immunosuppressive soluble factors within the tumor microenvironment dampens autophagy-related gene expression, potentially contributing to NK cell dysfunction in cancer.

### Autophagy modulation affects NK cell cytolytic activity

The protocols for allogeneic NK cell-based immunotherapies include ex-vivo expansion and cytokine activation. Since the ex vivo cytokine activation process influences autophagy flux (Fig. S5), confirming the reduction observed following IL15 exposure, we hypothesized that enhancing the autophagic machinery could modulate NK cell activity and thus their therapeutic potential.

**Fig. 4:**
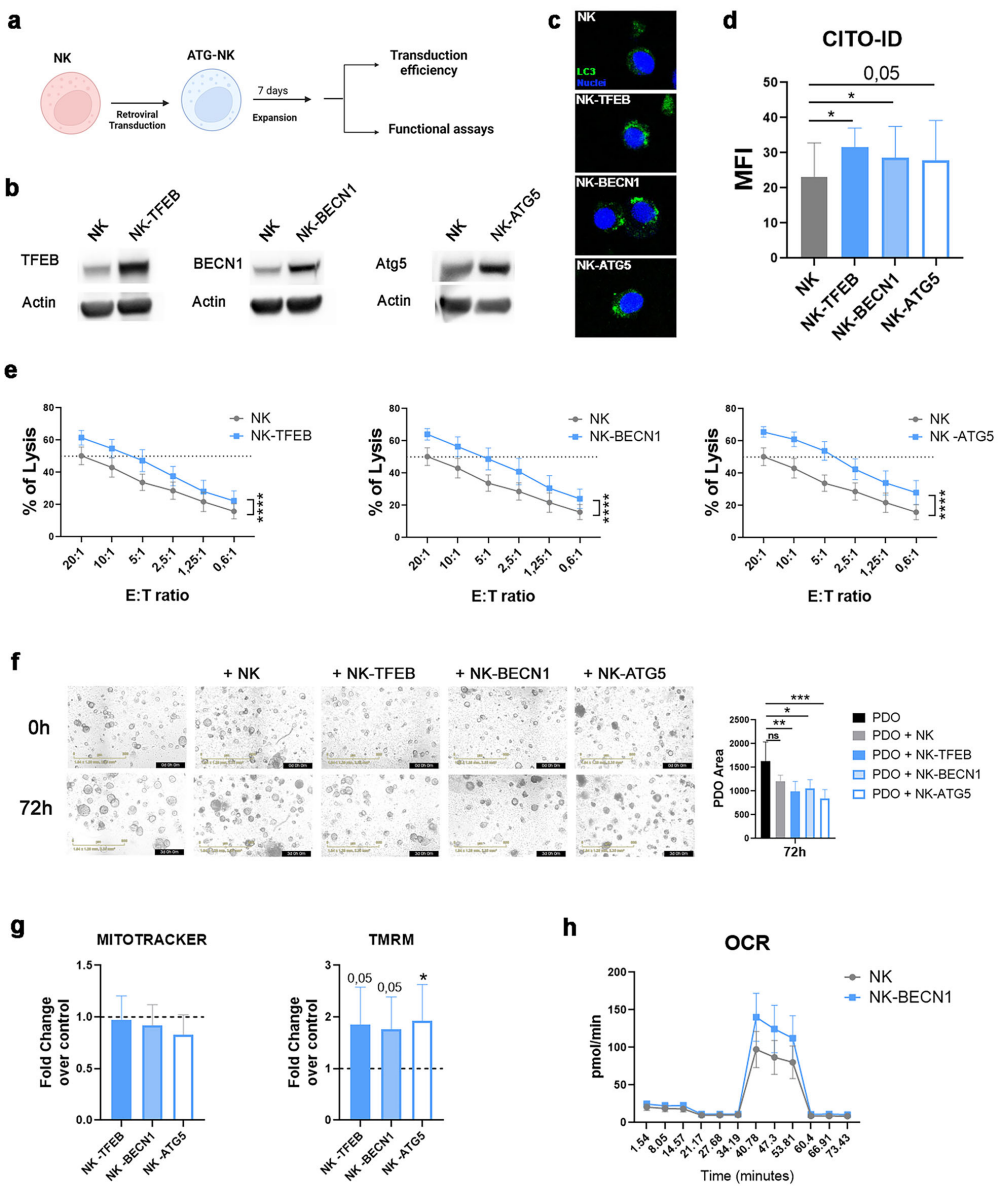
Overexpression of autophagy-related genes (ATG) modulates the cytotoxic activity of NK cells. **a** Schematic of experimental design. **b** Representative **i**mmunoblots of TFEB, BECN1, and ATG5 expression in NK cells 7 days post-transduction. **c** Representative images of LC3 staining. **d** Flow cytometry analysis of Median Fluorescence Intensity (MFI) of CYTO-ID staining. Data are means ± SEM of 5 biological replicates per condition. **e** NK cells were incubated with Nalm-18 tumor cells for 4 h at the indicated effector: target (E: T) ratio. Dead cells were evaluated as propidium iodide (PI)-positive cells by flow cytometry. Data are means ± SEM of 6 biological replicates per condition. **f** Representative images of co-cultured NK cells and pancreatic PDOs at 0 and 72 h. Bars represent the mean PDO area at 0, 24, 48, and 72 h of co-culture with mock or transduced ATG-NK cells from four healthy donors. **g** Mitochondrial mass and membrane potential state quantified by flow cytometry as the MFI of MitoTracker and TetraMethylRhodamine Methyl ester (TMRM). Data are represented as fold change of ATG-transduced NK cells over control NK cells. Data are means ± SEM of 5 biological replicates per condition. **h** Representative curve of Oxygen Consumption Rate (OCR) from one biological sample analyzed with the Seahorse XF analyzer using the Mito Stress Test kit. Statistical significance was assessed using Two-ways Anova test (**d**, **e** and **f**) and one-sample t-test (**g**) (*p < 0.05, **p < 0.01, *p* < 0.001***, *p* < 0.0001****).

To this end, we engineered NK cells to overexpress key autophagy regulators involved in different stages of the process: BECN1 (initiation), ATG5 (vesicle elongation), and TFEB (lysosomal biogenesis) (Fig. 4a). NK cells overexpressing autophagy regulators were referred to as ATG-NK cells. Control NK cells were transduced with an empty vector (NK). After 7 days, transduction efficiency was assessed through flow cytometric analysis of membrane staining for the CD19 reporter vector (Fig. S6a and b) and immunoblotting of the overexpressed gene (Figs. 4b and S6c). The increased presence of LC3 puncta and CYTO-ID staining confirmed higher autophagy levels in ATG-NK cells compared to NK cells (Fig. 4c and d). Subsequently, ATG-NK cells showed improved cytolytic activity against the acute lymphoblastic leukemia cell line Nalm-18 under cytokine-deprived conditions (Fig. 4e). Conversely, the presence of IL15 diminished this advantage (Fig. S7a). Pharmacological modulation using the autophagy inducer TAT-BECN1 or the inhibitor MRT-68921 [21, 22] further confirmed the role of autophagy in regulating NK cytotoxic function (Fig. S7b). However, the analysis of cytokine release showed no significant difference attributable to overexpression of autophagy-related genes (Fig. S8).

To evaluate the impact of autophagy in a three-dimensional (3D) model, mock- and ATG-NK cells were added to the culture medium of pancreatic tumor patient-derived organoids (PDOs) embedded in Matrigel. After 3 days of coculture, ATG-NK cells exhibited a higher ability to inhibit PDO growth than mock-NK cells, suggesting the role of ATG in NK cell cytotoxic activity and migration (Fig. 4f).

Autophagy is connected to cell metabolism fitness [23], which is essential for NK effector functions [24]. Therefore, to assess how autophagy modulation affects the metabolic machinery in NK cells, mitochondrial mass and membrane potential were measured. Although NK and ATG-NK cells showed similar mitochondrial mass, TMRM levels were higher in ATG-NK cells, indicating increased mitochondrial membrane potential (Fig. 4g). Additionally, the Oxygen Consumption Rate (OCR) was measured to compare oxidative phosphorylation (OXPHOS) between NK and BECN1 overexpressing NK cells (BECN1-NK cells) (Fig. 4h). The BECN1-overexpressing NK cells (BECN1-NK) exhibited higher basal and maximal OCR, spare respiratory capacity, and ATP production-linked respiration compared to untransduced NK cells (Fig. S9). Similar results were observed using NK cells overexpressing TFEB and ATG5 (Fig. S9). Together, these results suggest that autophagy improves mitochondrial and metabolic performance, providing a potential survival advantage and supporting NK cell effector functions.

### Autophagy supports the storage of cytotoxic granules in NK cells

**Fig. 5:**
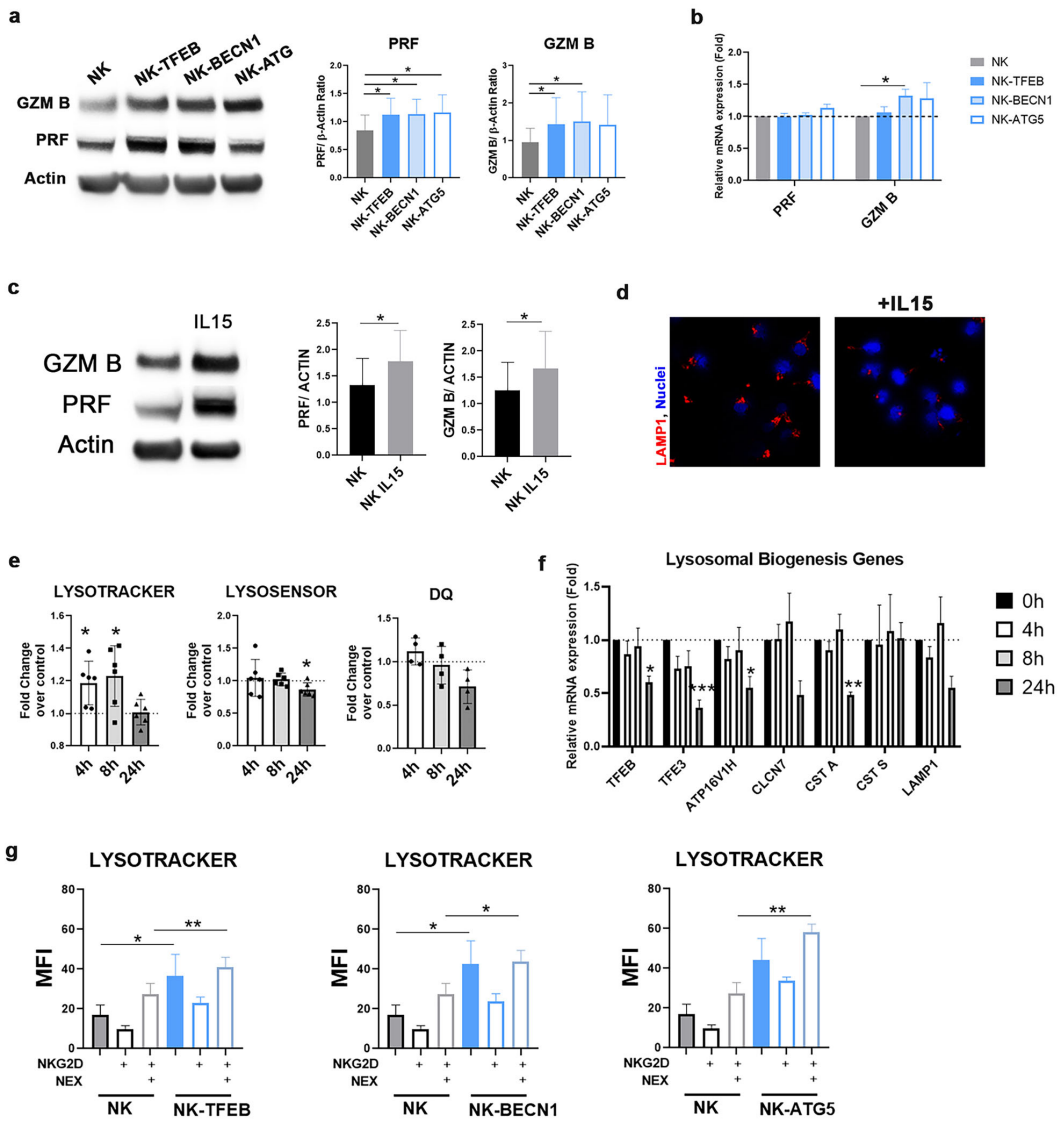
Autophagy enhances Perforin and Granzyme B availability in NK cells. **a** Representative immunoblot and quantification of GZM B and PRF protein levels in mock and ATG-NK cells cultured in IL15-starved conditions for 24 h. means ± SEM of 6 biological replicates (paired t-test; *p < 0.05). **b** Real-Time PCR analysis of GZM B and PRF transcripts normalized to 18S mRNA levels. means ± SEM of 4 biological replicates (paired t-test; *p < 0.05). **c** Representative immunoblot and quantification of GZM B and PRF protein levels in NK cells stimulated or not with IL15 for 24 h. means ± SEM of 6 biological replicates (paired *t* test; *p < 0.05). **d** Immunofluorescence staining for LAMP-1 after 8 h of IL15-starvation with anti-LAMP-1 (green) and Hoechst (nucleus, blue). **e** Lysosome mass, pH, and activity quantified by flow cytometry as the MFI of Lysotracker, Lysosensor, and DQ-BDA staining in IL15-starved NK cells at different time points. Data are represented as fold change of IL15-starved NK cells over IL15-stimulated NK cells (one-sample t-test, *p < 0.05). **f** Real-Time PCR analysis of lysosomal biogenesis gene transcripts normalized to 18S mRNA levels. means ± SEM of 4 biological replicates (Two-ways Anova test; *p < 0.05, **p < 0.01, *p* < 0.001***). **g** Flow cytometry analysis of Lysotracker staining of NK cells stimulated or not with NKG2D in the presence or absence of Nexhinib-20. Data are means ± SEM of 4 biological replicates per condition. Statistical significance was assessed using a paired t-test (*p < 0.05, **p < 0.01).

According to their enhanced cytotoxic activity, ATG-NK cells exhibited higher levels of granzyme B (GZMB) and perforin (PRF) compared to control NK cells (Fig. 5a). However, no increase in GZMB and PRF was observed in IL15-stimulated ATG-NK cells (Fig. S10). The GZM B and PRF mRNA levels were analyzed to assess their transcriptional regulation in ATG-NK cells. We observed similar levels of GZM B and PRF transcripts, except for GZM B in NK cells overexpressing BECN1 (Fig. 5b). These results indicate that the increase of cytotoxic protein was not fully explained by transcript level, suggesting a post-transcriptional mechanism, possibly due to enhanced lysosomal biogenesis and protein storage capacity likely linked to lysosome availability.

Lysosomes are the key organelles involved in autophagy, responsible for the degradation process. Conversely, autophagy also supports lysosome biogenesis by regenerating lysosomes from autolysosomes, a process called autophagic lysosome reformation (ALR) [25–27]. Therefore, we examined lysosomal mass and activity in NK cells during autophagy induced by IL15 starvation. IL15 stimulation increases GZM B and PRF protein levels (Fig. 5c). However, Lamp1 and LysoTracker staining revealed a significant increase in lysosomal mass during the first 8 h of IL15 starvation (Fig. 5d, e), with no changes in pH or lysosomal activity as shown by Lysosensor staining and the colorimetric DQ-BSA assay. Furthermore, the increase in lysosomal mass is not supported by higher expression of key genes involved in lysosomal biogenesis, indicating a potential role of autophagy in regulating lysosomal mass (Fig. 5f). Thus, we examined the lysosomal mass in NK cells overexpressing autophagy-related genes in resting conditions and during degranulation induction. NK cell degranulation was triggered by stimulating the activating receptor NKG2D using a monoclonal antibody. To observe lytic granule dynamics during degranulation, NK cells were either treated or untreated with the exocytosis inhibitor Nexhinib-20. After NKG2D stimulation, lysosomes were stained with the LysoTracker probe (Fig. 5g). As expected, NKG2D stimulation reduced lysosomal mass in both NK and ATG-NK cells, indicating the release of lytic granules. However, when degranulation was combined with Nexhinib-20 treatment, NK cells displayed increased LysoTracker staining intensity, indicating that lysosome mass modulation mainly depends on lytic granule release. Furthermore, ATG-NK cells exhibited higher LysoTracker staining intensity than control NK cells under both resting and degranulation conditions. These findings suggest that autophagy plays a role in the production of lytic granules during steady-states, which may provide an advantage during cytotoxic activity.

## Discussion

Autophagy is considered a potential therapeutic target in cancer treatment. However, its role in cancer remains controversial. While autophagy can prevent malignant transformation early on in tumor development, it often supports tumor cell survival and therapy resistance in advanced cancers [28–31]. Nevertheless, the precise relationship between autophagy and immune-mediated tumor control remains incompletely understood. In particular, despite the central role of NK cells in tumor immunosurveillance, the regulation and functional relevance of autophagy in human NK cells have remained largely unexplored. NK cells represent a first line of defense against cancer by directly eliminating transformed cells without prior antigen sensitization, and their antitumor efficacy critically depends on metabolic fitness, lysosomal integrity, and the availability of cytotoxic granules. Our study provides new insights into the role and regulation of autophagy in mature human NK cells and identifies autophagy as a key mechanism supporting NK cell cytotoxic potential under homeostatic conditions.

We show that the transcriptional profile of autophagy-related genes does not predict subset-specific autophagy potential in NK cells, indicating that autophagy is primarily regulated by environmental cues rather than intrinsic transcriptional programs. Indeed, autophagy is dynamically modulated by external stimuli, including cytokines (i.e., IL-15) and engagement of cytotoxic receptors. In particular, stimulation with IL-15, a cytokine essential for NK cell proliferation, survival, and effector function, suppresses autophagy. This result is consistent with previous studies showing autophagy suppression in CD8 T cells after exposure to inflammatory cytokines [32]. Although IL-15 enhances the proliferation, metabolic activity, and cytotoxic function of NK cells, prolonged exposure to IL-15 can cause NK cell exhaustion due to excessive mTOR signaling activation [33]. Therefore, it is reasonable to hypothesize that decreased autophagy may contribute to the NK cell exhaustion caused by IL-15 exposure. In contrast, during acute cytotoxic responses, autophagy inhibition is transient and rapidly compensated by transcriptional induction of autophagy regulators, suggesting the existence of adaptive mechanisms that restore cellular homeostasis following degranulation and target cell killing.

These findings may reflect receptor internalization and desensitization after prolonged exposure to ligand engagement, resulting in hyporesponsiveness of the activating receptor [34–36]. Furthermore, we observed a marked reduction in the transcriptional expression of autophagy regulators in NK cells exposed to malignant pleural effusions from lung cancer patients. Malignant pleural effusions contain low levels of nutrients and soluble factors released by immunosuppressive cells such as myeloid-derived suppressor cells (MDSC), tumor-associated macrophages (TAM), tumor-associated neutrophils (TAN), and regulatory T cells (Tregs). Therefore, these findings suggest that the tumor microenvironment (TME) actively suppresses the autophagy machinery in NK cells as an additional mechanism of immune evasion, potentially contributing to the well-documented metabolic impairment and functional exhaustion of tumor-infiltrating NK cells.

Notably, enhanced autophagy improves NK cell metabolism, granule biogenesis, and cytolytic activity in the absence of activating stimuli. This study proposes that autophagy maintains NK cell cytotoxic potential under homeostatic conditions by supporting mitochondrial fitness and lysosomal function, thereby ensuring rapid and effective responses upon activation. Autophagy supports mitochondrial fitness and lysosomal function, thereby ensuring rapid and effective responses upon encounter with target cells. Lysosomes were considered a static terminal compartment for degradative processes. Recent advances have revealed their involvement in nutrient sensing, metabolic adaptation, cellular stress handling, and exocytosis [37]. Lysosomes are dynamic organelles that shape their size by fission, fusion, new biogenesis, or recycling of autolysosome membranes [38, 39]. In NK cell education, structural remodeling of the endolysosomal compartment allows the accumulation of dense-core secretory lysosomes enabling the storage of granzyme B and enhanced cytotoxic activity in resting NK cells [40]. Educated NK cells increase Granzyme B levels independently of their transcription levels and of PI3K/AKT signaling triggered by cytokines and activating receptors. As expected, we showed that GZM B protein levels are lower in unstimulated NK cells compared to IL15-stimulated NK cells. However, deprivation of the activating cytokine IL15 increases both autophagy and lysosomal mass. The increase in lysosomal mass is not due to enhanced lysosomal biogenesis, indicating that autophagic lysosomal reformation might regulate lysosomal mass and preserve cytotoxic granules under cytokine-limiting conditions.

In conclusion, our findings identify autophagy as a critical intrinsic mechanism that sustains NK cell cytotoxic competence, particularly in resting or metabolically constrained environments such as the TME. Moreover, these results suggest that strategies aimed at preserving or enhancing autophagy in NK cells may improve their persistence, metabolic fitness, and antitumor efficacy in NK cell-based immunotherapies. Conversely, systemic inhibition of autophagy, currently explored as an anticancer strategy, may compromise NK cell-mediated tumor control. Thus, autophagy is important to maintain NK cell functionality and highlights autophagy modulation as a promising tool to enhance NK cell-driven anticancer immune responses.

## Methods

### NK-cell isolation and expansion

NK cells were obtained from the peripheral blood of healthy donors (HD) recruited by the Blood Transfusion Center of Bambino Gesù Pediatric Hospital. Overflow human bioptic samples were obtained and treated in accordance with the policies and practices of the Institutional Ethics Review Board of The OPBG Children’s Hospital, Rome (Prot. n°729/2023). Peripheral blood mononuclear cells (PBMC) were isolated after density gradient centrifugation (Ficoll-Lympholyte, Cederlane), and then NK cells were isolated by negative selection using RosettSep^TM^ Human NK Cell Enrichment Cocktail (StemCell). After isolation, NK cells were activated and expanded in MACSMix medium (Miltenyi) supplemented with 5% human serum and IL15 (150 U/ml, Miltenyi) and IL2 (500 U/ml, R&D, Minneapolis, MN, USA). Cells were maintained in a humidified atmosphere containing 8% CO_2_ at 37 °C. For the pharmacological modulation of autophagy, NK cells were incubated with 5 nM TAT-BECN1 (Selleck Chemicals) and 1 µM MRT-68921 (Selleck Chemicals) for 24 h.

### Cell lines

The Nalm-18 (Childhood B acute lymphoblastic leukemia) cell line was kindly provided by Dr Pende D. (IRCCS, Policlinico San Martino, Genoa, Italy) and cultured in RPMI 1640 (Euroclone, Milan, Italy) supplemented with 10% FBS (Gibco), 1% penicillin/streptomycin (Euroclone) and 1% L-glutamine (Euroclone).

### Retroviral transduction

Retroviral supernatant was generated in 293T-cells [41, 42] and quantified by Retro-X™ qRT-PCR Titration Kit (Takara). Viral supernatant was centrifuged at 2000 × *g* for two hours in 24-well plates pre-coated with recombinant human RetroNectin (Takara-Bio. Inc; Japan). After 5 days of culture in expansion medium, NK cells were seeded in virus-coated plates. Culture media were replaced after two days, and transduction efficiency was assessed by membrane surface staining (CD19).

### Sorting of NK-cell subsets

NK cells were first enriched from PBMCs by negative depletion and then stained with the fluorochrome-conjugated antibodies: CD56-PC7 (N901, Beckman Coulter), CD16-FITC (3G8BD, BD), CD3-PE-Vio610 (BW264/56, Miltenyi), and CD19-PE-Vio610 (LT19, Miltenyi). NK cell subsets were further purified by FACS cell sorting (CytoFLEX SRT Cell Sorter, Beckman Coulter): CD56bright NK cells were identified as CD56 + + /CD16-/CD3-/CD19- and CD56dim as CD56 + /CD16 + /CD3-/CD19-.

### Real-time PCR analysis

Total RNA extraction from NK cells was performed with RNeasy Micro kit (Qiagen) in compliance with the manufacturer’s protocol. RNA was reverse transcribed using High-Capacity cDNA Reverse Transcription Kit (Thermo-Fisher Scientific). Autophagy TaqMan® Array Card (Applied Biosystems) was loaded with TaqMan™ Fast Advanced Master Mix (Applied Biosystems). Real-time PCR was performed using PowerUp SYBR Green Master Mix (Applied Biosystems) on a QuantStudio 7 Flex instrument (Applied Biosystems).

Primer list:

**Table:** 

LC3	for CCCTCAGACCGGCCTTTCAA
	rev GATCACCGGGATTTTGCTGG
BECN1	for GGGCTCCCGAGGGATGG
	rev CTCGTGTCCAGTTTCAGGGG
ULK1	for CCACCCAGTTCCAAACACCT
	rev TCCACCCAGAGACATCTTCCT
Ambra1	for GAGCAGGATCCAGAGAGCAC
	rev CCTCTGGGCGTAGTATGCAG
GABARAP	for GGGCGAGAAGATCCGAAAGA
	rev TCCAGGTCTCCTATCCGAGC
ATG16L1	for CGAGGAGATCATCCTGCAATAAC
	rev CCTGTTTGGTACGTCATGCT
GZMB	for GATCATCGGGGGACATGAGG
	rev CCGCACCTCTTCAGAGACTT
Perforin	for ATTCCAGAGCCCAAGTGCC
	rev AGAAGGATGCCCAGGAGGAG

### Immunoblotting

NK cells were incubated in lysis buffer (65 mM Tris-HCl, pH 8, 1% NP40, 0.25% NaDOC, 150 mM NaCl, 1 mM EDTA, 0.1% SDS, protease-phosphatase inhibitor cocktail) for 20 min on ice. The samples were spun at 15,000 *g* for 15 min to remove insoluble components. The supernatant was collected as a whole-cell lysate. Cell lysate was heated at 85 °C for 2 min in 4X Bolt™ LDS Sample Buffer (Invitrogen) and loaded on self-cast polyacrylamide gels (gel percentages 13%) for LC3 detection and on precast Bolt 4–12% Bis-Tris Plus Gels (Invitrogen) for AKT, mTOR, GRZB, and Perforin detection. After SDS-PAGE, proteins were transferred to a nitrocellulose membrane (Amersham, GE Healthcare). The membranes were blocked in 5% nonfat dry milk (Cell Signaling Technology) in PBST (PBS containing 0.05% Tween 20) for one hour at room temperature (RT) and then incubated overnight at 4 °C with the following primary antibodies: anti-LC3 (Cell Signaling Technology mAb #3868), anti-AKT (Cell Signaling Technology mAb #9272), anti-phospho-AKT (Cell Signaling Technology mAb #4060), anti-mTOR (Cell Signaling Technology mAb #2983), anti-phospho-mTOR (Cell Signaling Technology mAb #2971S), Anti-β-Actin (Sigma-Aldrich A5441), anti-GZMB (Cell Signaling Technology mAb #4275S), anti-Perforin (Cell Signaling Technology mAb #6550S). The membranes were washed 3 times in PBST and incubated with HRP-linked secondary antibodies for one hour at RT (Cell Signaling Technology goat anti-rabbit IgG #7074 and goat-anti-mouse IgG #7076S). Signals were visualized using ECL Prime Western Blotting Detection Reagent (Amersham, GE Healthcare) on a Uvitec Cambridge Mini HD9.

### Flow cytometry sample analysis

The autophagic vacuoles were stained using the CYTO-ID® Autophagy Detection Kit (Enzo Life Sciences) according to the manufacturer’s guidelines.

Staining with Mitotracker Green (cat. No. M7514), MitoProbe TMRM (cat. No. M2003), and LysoTracker Deep Red (cat. No. L12492) was performed following the manufacturer’s protocols (Invitrogen).

To detect surface markers, NK cells were incubated for 20 min at 4 °C with the fluorochrome-conjugated antibody anti-CD19-FITC (clone LT19, Miltenyi).

Cells were acquired with the Beckman-Coulter Cytoflex-S flow-cytometer (Beckman-Coulter, Brea, CA, USA). A minimum of 5000 events for each condition were acquired. The acquired data were analyzed using CytExpert-2.4 (Beckman-Coulter) and FlowJo v.10 software (BD Biosciences).

### NK killing assay

The cytotoxic assay was performed as previously described [43]. Briefly, target cells were labeled with Green Cell Tracker (CMFDA; Invitrogen, Thermo Fisher Scientific) according to the manufacturer’s instructions and then incubated with NK cells at different effector/target ratios. After 4 h of incubation, propidium iodide (PI, Sigma-Aldrich) was added to detect dead tumor cells. Cells were acquired with the Beckman-Coulter Cytoflex-S/LX flow cytometer, and live target cells were identified as CMFDA + PI − , whereas dead target cells were CMFDA + PI + . Percentage (%) of cell lysis was calculated as follows: % cell lysis = ((% of dead cells cultured with NK) − (% of spontaneous lysis))/(100 − (% of spontaneous lysis)) × 100.

### Degranulation assay

The high-binding 96-well plate was incubated with Goat anti-mouse IgG (Jackson- ImmunoResearch Laboratories) at 4 °C overnight. The excess Goat anti-mouse IgG was then removed, and the plate was incubated with the anti-NKG2D antibody (clone BAT221, Miltenyi) at 37 °C for 15 min. After incubation, NK cells were seeded onto the NKG2D-coated plate and analyzed at multiple time points.

### 3D human pancreatic PDO culture and NK-mediated cytolysis assay

Patient-derived organoids were kindly provided by Prof. Dr. MD. med. Maximilian Reichert (Technical University of Munich (TUM), Germany). Generation of PDOs was approved by ethics review board of the TUM University Hospital, School of Medicine and Health, Technical University of Munich (Institutional Review Board project nos. 207/15, 1946/07, 330/19S, 181/17S, 5542/12 and 80/17S). PDOs were grown in Matrigel (Corning) domes in 24-well plates in feeding media containing: AdDMEM/F12 medium supplemented with GlutaMax (1X, Life Technologies), B27 (1X, Life Technologies), N2 (Life Technologies), N-acetyl-L-cysteine (1.25 mM, Sigma-Aldrich), Nicotinamide (10 mM, Sigma-Aldrich), recombinant human Wnt3a protein (100 ng/mL, R&D Systems), mNoggin (100 ng/mL, Preprotech), EGF (50 ng/mL, Life Technologies), Gastrin (10 nM, Sigma-Aldrich), FGF10 (100 ng/mL, Preprotech), and A83-01 (0.5 μM, Tocris). For passaging, the feeding medium was aspirated, and cold PBS was added to the well to dissolve the Matrigel matrix. The mixture of organoids, Matrigel, and PBS was transferred to a 15 mL tube and centrifuged. The organoid pellet was dissociated by enzymatic digestion with TrypLE (Life Technologies) at 37 °C for 10 min. After dissociation, the organoids were washed with cold PBS, mixed with Matrigel, and plated in a prewarmed 24-well plate. For the cytotoxic assay, PDOs were grown in a Matrigel matrix for 5 days, after which NK cells were added to the medium. NK cells and PDOs were cocultured for 72 h in the IncuCyte SX5 Live-Cell Analysis System. The assay plate was scanned every 24 h. The ability of NK cells to migrate toward and kill PDOs was evaluated by measuring the PDO area at multiple time points using ImageJ software.

### Evaluation of soluble cytokines, chemokines and growth factors in cell supernatants

We analyzed supernatants from mock-, TFEB-, BECN1-, or ATG5-transduced NK cells derived from 4 different HD. Transduced NK cells were cultured in RPMI 1640 (Euroclone, Milan, Italy) with or without IL-15 (150 U/ml, Miltenyi). After 48 h, supernatants were collected and analyzed in the same experiment, repeated twice using 25 μL. Levels of 36 soluble immune-related molecules were quantified using commercial MILLIPLEX Human Factor Panel A (Cat HSYTA-60K-36C, Millipore) according to manufacturer instructions. Samples were measured using Luminex technology on the instrument MAGPIX (Millipore) equipped with the xPONENT software, and data were expressed in pg/ml.

### Immunofluorescence staining and confocal microscopy

NK cells were seeded onto a coverslip and fixed in cold methanol for 5 min at −20 °C. The NK cells were washed three times with PBS, blocked for 1 h at RT with 20% goat serum, and incubated overnight at 4 °C with the primary antibody (anti-LC3 Cell Signaling Technology mAb #3868; anti-LAMP1 Cell Signaling Technology mAb #D4O1S). After three washes in PBS, the cells were incubated with fluorescently conjugated secondary antibodies, Goat Anti-Rabbit IgG Alexa Fluor 488 (Invitrogen) at room temperature for 2 h, washed three times with PBS, and stained with Hoechst. The coverslips were mounted on slides using VECTASHIELD HardSet Antifade Mounting Medium (Vector Laboratories). Images were taken using a Leica SP8.

### Metabolic assay

NK cells were seeded in Seahorse poly-D-lysine-coated Miniplates. The oxygen consumption rate was measured using the Seahorse XF HS Mini Analyzer with the Mito Stress Test kit, following the manufacturer’s guidelines (Agilent).

### CITE-Sequencing data analysis

CITE-Seq data from the GEO dataset GSM5008737 were analyzed using custom Python-based scripts based on Scanpy and scvi-tools [44, 45]. Quality control metrics were computed with the *calculate_qc_metrics* function in Scanpy. Cells were filtered based on multiple criteria: log-transformed total counts and the number of detected genes per cell were evaluated, and outliers exceeding ± 5 median absolute deviations (MADs) from the median were removed. Additionally, cells with more than 5 MADs above the median percentage of counts in the top 20 most expressed genes were flagged as outliers. Mitochondrial content was assessed separately: cells with mitochondrial gene expression exceeding 3 MADs from the median or with more than 8% mitochondrial reads were excluded. Following filtering, cells expressing fewer than 200 genes and genes detected in fewer than 3 cells were also removed. Scrublet was used to identify and remove predicted doublets (PMID: 30954476). Data normalization was performed using total count normalization (CPM × 10,000) with Scanpy’s *normalize_total* function, followed by a log1p transformation. Sample metadata, including donor and cell type (celltype.l2), were mapped to the AnnData object. Similarly, ADT counts were loaded and aligned with RNA data based on common cells. A MuData object was created by combining RNA and protein data, with modalities named “rna” and “protein”. Batch-aware highly variable gene selection was performed using the *highly_variable_genes()* function, setting the *batch_key* to “donor”. A TOTALVI model was trained using RNA counts and protein data, with “donor” as the batch key. Dimensionality reduction was performed on the latent space (*X_totalVI*) via neighborhood graph and UMAP (*n_neighbors* = *10, min_dist* = *0.1, spread* = *0.8*). NK cells were identified based on cell type annotations from Hao et al. [11] and analyzed separately. Clustering resolution was optimized by calculating the silhouette score, identifying 0.3 as the optimal value. Resulting clusters were annotated into NK cell subsets (i.e., CD56bright, CD56dim cytotoxic, and CD56dim memory). Differential gene expression was computed using a Wilcoxon test, and marker genes were visualized via dot plots before and after filtering for cluster specificity. To visualize autophagy gene expression across NK cell subsets, we used the Scanpy function *sc.pl.dotplot*, where the dot color represents the average expression and the dot size indicates the percentage of cells expressing the respective autophagy gene. The autophagy score for each cell was calculated using *scanpy.tl.score_genes*, based on the average expression of the genes in the Human Autophagy Database (HADb). This score was then visualized on the UMAP embedding to examine its distribution among cell populations.

### Single-cell RNA-sequencing data analysis

Single-cell RNA sequencing (scRNA-seq) data from the ArrayExpress dataset E-MTAB-13204 [46] were analyzed to assess the interaction of NK cells with tumor cells at various time points. The dataset included two multiplexed experimental plates containing both gene expression and Hashtag Oligonucleotide (HTO) libraries. Data from two experimental plates were integrated to create a unified Seurat object for downstream analysis. For each plate, raw gene expression and HTO count matrices were imported and converted into Seurat objects. HTO-based sample demultiplexing and initial filtering were performed using Seurat (v5.3.0) in R. Mitochondrial and ribosomal gene percentages were computed, and cells were filtered based on quality control thresholds: mitochondrial content <15%, ribosomal content between 5–50%, total RNA counts (nCount_RNA) > 700, and the number of detected genes (nFeature_RNA) between 300 and 10,000. HTO counts were normalized using the centered log-ratio (CLR) transformation, and demultiplexing was performed with the HTODemux function (positive quantile = 0.999). Only cells classified as “Singlet” were retained, while “Doublet” and “Negative” events were discarded. Each “Singlet” cell was assigned to its corresponding biological sample by matching the detected HTO barcode to an external metadata table provided by Dufva et al. [46]. To ensure unique cell identifiers across datasets, cell barcodes were prefixed with “Plate1_” or “Plate2_”, and the metadata tables were merged accordingly. A batch variable was added to the metadata to track the plate of origin. Following data integration, raw counts were normalized by scaling each cell’s library size to 10,000 total counts and applying a with log-transformation (log1p). This normalization was applied uniformly across all cells. Variable gene selection was performed using the “mean.var.plot” method, selecting the top 2,000 most variable features. To account for technical and biological confounders, cell cycle scores were computed using Seurat’s built-in cc.genes set, and scaling was performed while regressing out mitochondrial content, total RNA count, batch, and cell cycle phase scores. Cell types were annotated using the SingleR package, leveraging the *DatabaseImmuneCellExpressionData* reference dataset. NK cells were identified based on the assigned SingleR labels and extracted for downstream analysis. A curated list of autophagy-related genes (AMBRA1, ULK1, BECN1, ATG16L1, ATG7, ATG10, MAP1LC3B, GABARAP, GABARAPL1, GABARAPL2, NBR1, SQSTM1, PINK1, WDR45, and WIPI1) was compiled and filtered to retain only those expressed without missing values. Expression of these genes was visualized using dot plots (DotPlot) grouped by sample identity. Specifically, NK cells were grouped based on the target tumor cell line (K562, GDM1, JURKAT, RI1) and time point (1–24h). For each condition, the change in mean normalized expression (computed as the difference from the baseline NK_0h mean log-normalized value) and the percentage of expressing cells were calculated. These metrics were visualized in custom dot plots, where color encodes the difference in the mean expression and dot size indicates the proportion of cells expressing the respective ATG. All visualizations were generated using ggplot2, patchwork, and cowplot packages.

### Statistical analysis

The sample size used is indicated in the legend of each figure. Quantitative data are presented as the means ± SDs or means ± SEMs. Statistical analysis was performed with Prism 8 (GraphPad Software, San Diego, Calif). Normality was assessed using the Shapiro-Wilk test. The Mann-Whitney test or Student’s *t* test was used to determine statistical significance. Differences were considered significant at *p* < 0.05.

### Ethics approval and consent to participate

All methods were carried out in accordance with relevant guidelines and regulations. Informed consent was obtained from all participants or their legal guardians.

## Supplementary information

Supplemental Material

Original Data

Original Data supplementary

## Data Availability

All data relevant to the study are included in the article or uploaded as online supplementary information. Data or further information is available from the corresponding author upon reasonable request.
