# Supplementary material for: Autophagy supports the storage of lytic granules in human NK cells
Source: Cell Death Dis. 2026 Jun 1;17(1):684. doi: 10.1038/s41419-026-08937-1 (PMC13439192; doi:10.1038/s41419-026-08937-1)
Supplement: Supplementary file 1 [file 41419_2026_8937_MOESM1_ESM.docx]

S1. Analysis of the CITE-seq dataset of NK cells isolated from PBMCs of healthy donors (HD). **a,** Dot plot of the 20 most distinguishing proteins expressed in the indicated NK cell populations. **b**, Dot plot of the 20 most distinguishing genes expressed in the indicated NK cell populations.

**S2**. Modulation of autophagy regulation in NK cells isolated from the peripheral blood (PB) of healthy donors (HDs) and stimulated with IL15 for 24h. **a,** Z-score heatmap showing the RT-qPCR array analysis of autophagy regulator genes in pbNK cells upon IL15 stimulation. Bars represent the statistical significance of the differences between unstimulated and IL15-stimulated NK for each gene as -log_10_ transformed t-test p-values (green: p-value$\leq$0.05, purple: p-value>0.05). **b**, Representative Immunoblot and quantification of the LC3 I and LC3 II ratio changes in unstimulated and IL15-stimulated pbNK cells. Pb-NK cells were treated with 10µM Chloroquine (CQ) before immunoblotting. **c**, Representative Immunoblots and quantification of total and phosphorylated mTOR, and ACTIN in unstimulated and IL15-stimulated pbNK cells. Data are means ± SD of 4 to 6 biological replicates per condition. p-values were calculated using a paired t-test (*p<0.05, **p<0.01).

S3. Representative immunoblot and quantification of BECN1 and Bcl-2 in unstimulated and IL15-stimulated NK cells isolated from the peripheral blood of healthy donors (pbNK) and ex vivo expanded NK cells (NK). p-values were calculated using a paired t-test (*p<0.05, **p<0.01).

**S4** Analysis of scRNA-seq datasets of NK cells and tumor cell cocultures. Custom dot plot showing the change in mean normalized expression (relative to baseline, 0h) in ATG expression in NK cells at different time points after co-culture with Jurkat, RI1, and GDM1 cell lines. The x-axis shows the ATG names, and the y-axis indicates the stimulation time point. The dot color represents the average expression changes, and the dot size corresponds to the percentage of cells expressing the respective ATG.

**S5** Autophagy modulation in NK cells isolated from the peripheral blood of healthy donors (pbNK) and ex vivo expanded NK cells (exNK). **a**, Real-Time PCR analysis of ULK1 and LC3transcripts normalized to 18S mRNA. Data are presented as means ± SD of 5 biological replicates per condition. p-values were calculated using a paired t-test (*p<0.05, **p<0.01). **b**, Representative Immunoblot and quantification of the LC3 I and LC3 II ratio changes. NK cells were treated with 10µM Chloroquine (CQ) before immunoblotting.

**S6.** Overexpression of autophagy-related genes (ATG) in NK cells. **a**, Flow cytometric assessment of gene transfer by immunostaining of CD19 after 7 days of transduction. **B,** Percentages of CD19-positive cells at different time points after transduction. The results are the means ± SD of eight different NK cell donors. **c**, Densitometer-intensity data of **i**mmunoblots of TFEB, BECN1, and ATG5 expression. Mean ± SD of 5-6 experiments. p-values were calculated using a t-test (*p<0.05, **p<0.01).

**S7.** Modulation of autophagy affects the cytolytic activity of unstimulated NK cells. **a**, Cytotoxicity assay with IL15-stimulated transduced NK cells against NALM-18 target cells. Transduced NK cells were incubated with Nalm-18 tumor cells for 4h at the indicated E: T ratio. Dead cells were evaluated as propidium iodide (PI)-positive cells by flow cytometry. Mean of 6 independent experiments ± SEM. p-values were calculated using a t-test (*p<0.05, **p<0.01). **b**, NK cells were treated or not with autophagy inducer TAT-BECN1 or MRT 68921 for 24h in the presence (left panel) or absence (right panel). After treatment, a cytotoxicity assay was performed against NALM-18 cells. Mean of 4-5 independent experiments ± SEM. Statistical significance was assessed using Two-ways Anova test (*p<0.05, **p<0.01, p<0.001***,p<0.0001****).

S8. Cytokine release analysis of ATG-transduced NK cells. The supernatants from ATG-NK and mock-NK cells cultured for 48h with or without IL15 were analyzed using Luminex multiplex assays. The results were presented as a heatmap of soluble cytokine concentrations. Mean of 4 independent experiments.

**S9.** Overexpression of autophagy regulator genes improves mitochondrial fitness in NK cells. Oxygen Consumption Rate (OCR) was measured by Seahorse XF analyzer with the Mito Stress Test kit. The results are the mean of 5 out to 6 experiments ± SD. p-values were calculated using a two-sample t-test (*p<0.05).

**S10.** Perforin and Granzyme B (GZM B) expression in IL15-stimulated NK cells. Representative immunoblot and quantification of Granzyme B, Perforin, and Actin. Data are means ± SEM, 6 biological replicates per condition.
